# An International Survey-based Algorithm for the Pharmacologic Treatment of Chorea in Huntington’s Disease

**DOI:** 10.1371/currents.RRN1260

**Published:** 2011-10-11

**Authors:** Jean-Marc Burgunder, Mark Guttman, Susan Perlman, Nathan Goodman, Daniel P. van Kammen, LaVonne Goodman

**Affiliations:** ^*^Department of Neurology, University of Bern, Switzerland; ^†^Division of Neurology, Department of Medicine, University of Toronto, Toronto, Ontario Canada; ^‡^David Geffen School of Medicine at UCLA; ^§^Institute for Systems Biology, Seattle, WA; ^¶^Formerly CHDI Foundation, Inc. Presently independent CNS development consultant and ^#^Huntington's Disease Drug Works, Lake Forest Park, WA

## Abstract

It is generally believed that treatments are available to manage chorea in Huntington’s disease (HD). However, lack of evidence prevents the establishment of treatment guidelines. The HD chorea research literature fails to address the indications for drug treatment, drug selection, drug dosing and side effect profiles, management of inadequate response to a single drug, and preferred drug when behavioral symptoms comorbid to chorea are present. Because there is lack of an evidence base to inform clinical decision-making, we surveyed an international group of experts to address these points. Survey results showed that patient stigma, physical injury, gait instability, work interference, and disturbed sleep were indications for a drug treatment trial. However, the experts did not agree on first choice of chorea drug, with the majority of experts in Europe favoring an antipsychotic drug (APD), and a near equal split in first choice between an APD and tetrabenazine (TBZ) among experts from North America and Australia. All experts chose an APD when comorbid psychotic or aggressive behaviors were present, or when active depression prevented the use of TBZ. However, there was agreement from all geographic regions that both APDs and TBZ were acceptable as monotherapy in other situations. Perceived efficacy and side effect profiles were similar for APDs and TBZ, except for depression as a significant side effect of TBZ. Experts used a combination of an APD and TBZ when treatment required both drugs for control of chorea and a concurrent comorbid symptom, or when severe chorea was inadequately controlled by either drug alone. The benzodiazepines (BZDs) were judged ineffective as monotherapy but useful as adjunctive therapy, particularly when chorea was exacerbated by anxiety. There was broad disagreement about the use of amantadine for chorea. Experts who had used amantadine described its benefit as small and transient. In addition to survey results, this report reviews available chorea studies, and lastly presents an algorithm for the treatment of chorea in HD which is based on expert preferences obtained through this international survey.

## **Introduction**

Huntington’s disease (HD) is a progressive neurodegenerative disorder transmitted by an autosomal dominant inheritance via an elongated CAG nucleotide repeat on chromosome 4 [1].  At present, there are no established therapies which have been shown to delay onset or alter progression of this disease.  In addition to cognitive impairment and a wide variety of psychiatric features, HD is characterized by a combination of complex hyper-and hypokinetic motor syndromes that vary among affected individuals, and change over the stages of disease within a single individual. Hyperkinetic motor signs include involuntary chorea movements, which peak in early and mid-stage disease in adult onset individuals, and subsequently decline as the disease progresses. Dystonia is another involuntary motor disorder which occurs predominantly in later stage disease.  Bradykinesia, or paucity of movement, is a voluntary motor impairment that is present very early in the course of disease and progresses steadily to end-stage akinetic rigidity.  Except for the use of Botox for isolated dystonic reactions, chorea is the only motor symptom for which there are therapeutic options. 

Age of onset in HD can vary from early childhood [2] to advanced age [3], but most commonly occurs between the ages of 30 and 50 years.  After onset the disease follows a progressive degenerative course with an average duration of approximately 20 years, when age of onset is between 20 and 50 years of age [4].  Chorea is an early and highly visible sign of the disease in approximately 90% of adult-onset HD patients, peaking at about 10 years after first emergence of this symptom, then gradually abating as the disease progresses.  Chorea is mild or absent in juvenile onset disease and in 10% of adult onset patients.  The pattern of choreic movements differs among individuals and can include facial pouting, grimacing, and lifting of alternate eyebrows; forward, backward, and rotational neck and trunk movements, upper and lower extremity asymmetric flexion or extension of both small and larger muscle groups, and frequent crossing of the arms and legs [5].  The gold standard definition for onset of HD remains an open debate (plos)  Although it is now known that cognitive and behavioral symptoms may predate onset of motor signs in many patients [6], onset, as defined in PREDICT-HD is that point in time when the investigator is confident in the diagnosis of unequivocal motor signs.  However, in clinical practice, due to its high visibility. chorea is the motor sign most often used as the clinical marker that defines onset of the disease in adults [7].   

**Box 1. Abbreviations for drugs and drug classes**

**Table:** 

AED	mood stabilizing anti-epileptic drug
APD	antipsychotic
BZD	benzodiazepine
SSRI	selective serotonin reuptake inhibitor
TBZ	tetrabenazine

 It is generally agreed that drugs are available in clinical practice that can improve chorea [8] [9] [10].  However, as summarized in the 2009 Cochrane review “Therapeutic intervention for symptomatic treatment in Huntington’s disease,” there is a lack of sufficient efficacy studies for individual drugs, and lack of drug comparison studies to obtain clear clinical guidance regarding drug choice.  The authors cite TBZ as the antichoreic medication with best available clinical evidence; but they were unable to give any specific recommendation about best medical practices for treating chorea [11].  Lacking an adequate evidence base, we surveyed an international group of HD experts to ascertain practice-based preferences.  Within the limits of expert opinion, and with the expectation that future clinical research will provide better information, we present survey results, and propose an algorithm to guide the clinical management of chorea in HD.  The goal of the algorithm is to concisely deliver relevant and expert knowledge to point-of-care medical providers, and to update when evidence-based recommendations emerge. 

## **Methods**

This chorea survey was one of three symptom surveys developed by 2 core groups of nine psychiatrists and neurologists drawn from the European Huntington’s Disease Network (EHDN) and the Huntington Study Group (HSG), and a HD family representative.  Concurrent surveys were developed for obsessive compulsive behaviors and irritability symptoms in HD.  These specific three symptoms were chosen as those in greatest need of expert guidance relative to other symptoms of HD, including depression, anxiety, sleep disorder, and psychotic behaviors, for which clinical practice follows guidelines developed for these conditions in the general population.  Data on concurrent surveys for the treatment of irritability and obsessive compulsive behaviors is presented in separate reports [12] [13].

 Three neurologists from different geographic areas who had extensive experience treating chorea in HD, and an HD family member developed the chorea survey.  Individual questions were framed to gather information on clinicians’ indications for drug treatment, drug selection, dosing, management of inadequate drug responses, treatment of side effects, and management of chorea when complicated by concurrent symptoms of the disease.  Survey questions were developed electronically on software that utilized branching logic in queries. The survey was subsequently distributed by email link to a larger international group of EHDN and HSG physician leaders from HD specialty centers in 11 European countries, 10 U.S.A states, 4 Canadian provinces, and 3 Australian states.  Experts were selected by the combined EHDN and HSG core group members as being knowledgeable in treating HD symptoms.  Follow-up email or telephone reminders were used to encourage survey participation. 

The initial chorea survey contained 28 multiple choice questions with 168 alternative answers, with the option to add additional comments. Questions addressed respondents' demographics, clinical indications for treating chorea, and patterns of pharmacologic treatment  By core group consensus, the survey focused on 2 drug classes (APDs and BZDs) and 2 drugs (TBZ and amantadine) that have been used to treat chorea.   In  iterative fashion, each medication class was addressed separately through additional questions covering the following: patterns of use (first choice, alternative monotherapy, adjunctive therapy, not an appropriate use, insufficient experience), perceived effectiveness (very effective, effective, somewhat effective, minimally effective), preferred drugs within each class, and side effect profiles.  Branching logic utilized in the electronic survey prevented the answering of questions if a respondent did not choose a specific treatment as first or alternative monotherapy, or indicated having no experience with a particular treatment, Questions also covered dose titration, preferred APDs, and preferred drug for chorea when comorbid behavioral symptoms were present.  Due to an omission in the original survey that became apparent during data analysis, we distributed an addendum to the original survey, presenting 3 additional questions with 10 alternative answers specifically addressing the combination of APDs and TBZ.  After completion of the first part of the survey, respondents received a small honorarium. 

 Following analysis of survey data, we presented an algorithm for the treatment of chorea at the Spring 2010 EHDN conference and the Fall 2010 HSG symposium for final review by a broader group of international experts.   

## **Results**

The original survey was sent to a total of 66 international expert physicians, of whom 52 (79%) completed at least part of the survey.  Respondents were predominantly from the United States of America (N=24) and Europe (N=22), with a few others based in Canada (N=4) and Australia (N=2). The majority of respondents were boarded in Neurology (N=41), psychiatry (9), or double-boarded in Neurology and Psychiatry (N=2). Three of nine psychiatrist respondents cited limited experience in treating chorea. Among the remaining 49 responders who answered the majority of questions, clinical experience was quite substantial: over half of the respondents reported treating more than 100 HD patients annually.  The survey addendum was sent to the 49 respondents from the original survey, of whom 30 (61%) responded.

**Treatment indications:  **Respondents were first queried about factors that would warrant drug treatment of HD chorea.  The most noted indications were stigma factors of patient embarrassment and social isolation, physical injury, loss of balance, and interference with employment or sleep.  Interference with caregiver tasks rated highly, but family stigma factors of embarrassment and social isolation were not considered as compelling as treatment indications. 

**Table 1. Percentage of respondents choosing specific indications for drug treatment of chorea.**

**Table:** 

**Indications for drug treatment of chorea**	**Respondents affirming indication (%)**
Patient embarrassment	92%
Physical injury due to chorea	88%
Loss of balance	81%
Interference with caregiver tasks	79%
Social isolation	77%
Interference with work	77%
Interference with sleep	64%
Family embarrassment	32%

**Practice patterns by drug or drug class:  **The first set of treatment questions concerned drug selection and was phrased as follows: “Assuming there are no comorbid symptoms to influence your decision, what is your practice pattern with the use of [drug or drug class] for the treatment of chorea in Huntington’s disease?”  In iterative manner for each separate query on drug or drug class, respondents were asked to choose either (1) first choice, (2) alternative monotherapy, (3) adjunctive therapy, or (4) inappropriate, (5) insufficient experience.   Interpretation of results for this set of questions was complicated by limitations inherent in survey software, which allowed the respondent to check more than one first choice, or to check no first choice.  An additional complicating factor for this set of questions was that respondents were asked to consider chorea as an isolated symptom, which is an uncommon situation in real-world clinical practice, where comorbid symptoms strongly influence choice of chorea drug.  See figures 1-3 and table 2.

**Figure:**
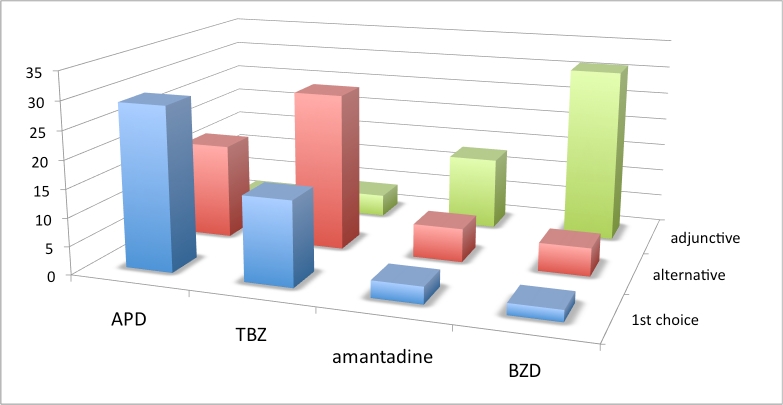
**Figure 1. Choice of chorea drug across all geographic regions. Vertical axis is number of responses. See box 1 for abbreviations.**

**Figure:**
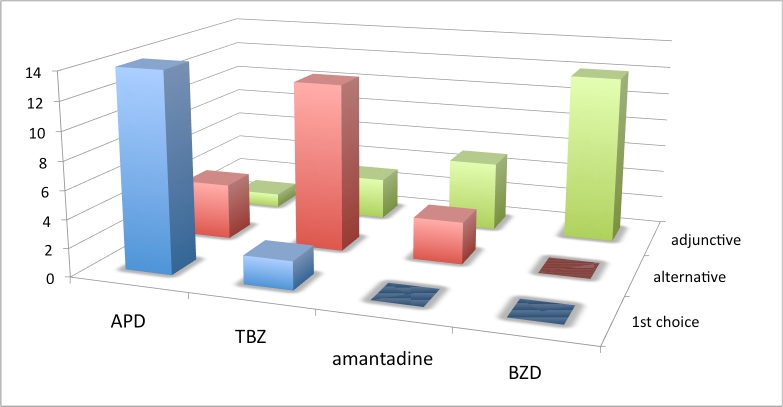
**Figure 2. Choice of chorea drug, European respondents. Vertical axis is number of responses.  See box 1 for abbreviations.**

**Figure:**
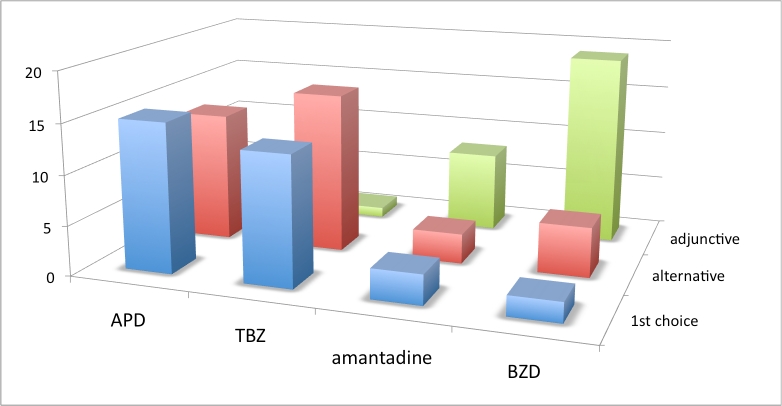
**Figure 3. Choice of chorea drug, North American and Australian respondents.  Vertical axis is number of responses.  See box 1 for abbreviations.**

**Table 2. Choice of drug for treating chorea across all geographic regions. N is number of responses. Percentages are relative to N. See box 1 for abbreviations.**

**Table:** 

**Drug or drug class**	**N**	**First choice**	**Alternative monotherapy**	**Adjunctive therapy**	**Inappropriate**	**Insufficient experience**
**APD**	50	58%	34%	4%	2%	2%
**TBZ**	50	30%	56%	8%	0%	6%
**BZD**	50	6%	12%	26%	22%	34%
**amantadine**	50	4%	10%	62%	22%	2%

Results from this set of questions showed a lack of consensus about the first choice of chorea drug when analyzed across all geographic regions.  APDs were the first choice of most European and North American respondents.  However, due to limitations when using survey branching logic, 15% of respondents checked more than one first choice, and an equal number checked no first choice.  We speculate that these clinicians had discomfort with a single first choice preference.  Results also show a striking variation of first-choice drug across geographic regions.**  **Although APDs were the first choice of the majority of both European and North American respondents, a greater number of HSG respondents from North America and Australia (43%) chose TBZ as first-choice drug, compared to only 11% of EHDN European respondents. 

When analysis of combined monotherapy choices (first and alternative) was performed, APDs scored only slightly higher (92.3%) than the combined numbers for TBZ (86.5%).  Benzodiazepines were chosen as adjunctive therapy.   However, respondents disagreed about the use of amantadine:  A minority considered it useful as monotherapy or adjunctive therapy, while a smaller but not insignificant number of respondents considered its use inappropriate for the treatment of chorea, and several cited inexperience with its use.  

**Perceived effectiveness of drug choice:  **Ratings for the perceived effectiveness of APD and TBZ for HD chorea were similar for these two treatment options.  Experts rated APDs for the treatment of chorea as follows:  “very effective” (20%), “effective” (64%), or “somewhat effective” (16%).  No respondent perceived APDs as ineffective.  Experts rated the perceived effectiveness of TBZ as follows: “very effective” (20%), “effective” (48%), and “somewhat effective” (28%).  In contrast, most respondents who used either BZD or amantadine rated both medications as only “somewhat” or “minimally” effective as monotherapy.

**Preferred APD monotherapy for HD chorea: **There was wide variation in APD choice. Olanzapine and risperidone were most preferred.  Tiapride, a first generation neuroleptic not available in North America, was preferred by 50% of European respondents.  Again, due to survey software limitations, several respondents checked more than one first choice.  Regarding APD dosing for chorea, though specific dose range for individual drugs was not addressed, 92% of respondents preferred APD dosing lower than manufacturer recommended recommendations for treating psychosis.

**Table 3. APD of first choice (all geographic areas). APD listed only if chosen by more than two respondents.**

**Table:** 

**APD of first choice**	**Respondents reporting (%)**
risperidone	43%
olanzapine	39%
tiapride*	29%
haloperidol	24%
quetiapine	12%
aripiprazole	11%

*Tiapride only available in certain European countries.   

**Preferred BZD for adjunctive therapy:  **Clonazepam was the preferred BZD for adjunctive treatment of chorea by 79.5% of respondents, with several citing its lower addictive potential compared to other BZDs.   Lorazepam was chosen by a minority (12.5%) of respondents.

**TBZ specific practice:  **Several questions focused specifically on TBZ, including contraindications, interval titration of dosage, frequency of depression as a side effect, and treatment strategy for TBZ-associated depression. Regarding contraindications: the great majority of respondents (90%) would not use TBZ with concurrent active depression, but indicated that past or treated depression was not a contraindication for use (88%).  Given a past history of suicidal gesture, 24% would not use TBZ, while a larger fraction (48%) would not use the drug if a suicide attempt had been made at any time in the past.  Regarding dose titration intervals: the minority of respondents (16%) chose the 1-2 week titration interval recommended by Lundbeck Inc., distributor of the drug in the United States.  More respondents (50%) chose a 2-4 week titration interval, and 26.5% chose an interval of more than 4 weeks.  Regarding TBZ-associated depression: respondents reported the perceived frequency of occurrence as follows: “very frequently” (4%), “frequently” (14%), “somewhat frequently” (36%), and “infrequently” (38%).   When depression occurred with TBZ, 26% of respondents decreased TBZ dosage, while 10.2% added or increased dosage of an antidepressant without changing TBZ dosage. A larger number of respondents (34.7%) did both: decreased TBZ dosage and added or increased antidepressant therapy.  A minority of respondents (20%) discontinued TBZ.   When depression occurred, most respondents (69.5%) reported that it was more likely to occur in the first two months of TBZ therapy, but many cited lack of long-term experience with TBZ. 

**Frequency of perceived side effects due to APDs or TBZ:  **Respondents rated the frequency of observed side effects associated with APDs and TBZ (Table 4). When comparing APDs and TBZ, experts reported that sedation and cognitive decline appeared to occur at similar frequencies (less than a 3% difference).  Apathy, Parkinsonism, metabolic syndrome, and tardive dyskinesia side effects were perceived to be higher for APDs than for TBZ.  However, depression was frequently cited as a side effect of TBZ.  Akathisia (motor or psychic restlessness) was reported as a side effect for both types of agent at similar frequencies. Due to an error of omission in the survey queries, swallowing disorder associated with APDs and TBZ could not be compared.

**Table 4. Side effects reported as occurring "very frequently" or "frequently" with APDs and TBZ. See box 1 for abbreviations.**

**Table:** 

**Side effect**	**Drug or drug class**	**Respondents reporting (%)**
**Sedation**	APD	48%
	TBZ	46%
**Depression**	APD	Not queried
	TBZ	26%
**Parkinsonism**	APD	18%
	TBZ	13%
**Apathy**	APD	18%
	TBZ	13%
**Cognitive impairment**	APD	10%
	TBZ	10%
**Swallowing disorder**	APD	Not queried
	TBZ	4%
**Akathisia (motor and psychic restlessness)**	APD	10%
	TBZ	8%
**Metabolic syndrome**	APD	8%
	TBZ	Not queried
**Tardive dyskinesia**	APD	4%
	TBZ	0%

**First choice of drug when comorbid symptoms are present:**  Though the majority of survey questions concerned the treatment of chorea as an isolated symptom, it is more common for HD patients to present with multiple symptoms that may influence choice of drug.   APDs were the universal first-choice drug for treating chorea when it occurs in the setting of comorbid symptoms of psychotic or aggressive behaviors, and active depression. If poor compliance was suspected, APD was preferred over TBZ.

**Combining APDs and TBZ:**  Following the original survey, additional information was solicited about the clinical practice experience of combining APDs with TBZ.  Though a smaller number (32) of the original {49) respondents who cited experience with treating chorea responded to the appended question, most of this subset (75%) had experience combining APDs and TBZ.  The majority of those who had used the combination (80%) cited the need to control chorea and concurrent comorbid symptoms.  A smaller fraction (40%) had used the combination to treat severe chorea not adequately controlled by a single drug. As for each agent independently, experts combining APDs and TBZ reported Parkinsonism, apathy, and akathisia as the most common side effects.  

## **Discussion**

**Indications for drug treatment:** Drug treatment decisions for HD chorea have the goal of improving quality of life.  However in practice, assessing the benefit of treatment on quality of life is difficult because there have been no chorea studies that have used quality of life as a measure, and lack of studies on chorea’s impact on stigma or motor dysfunction. Further, assessing side effects of chorea drugs, which include apathy, Parkinsonism, and worsening cognition are difficult to separate from signs of disease progression. There has also been general disagreement about the impact of chorea, citing the lack of patient awareness [14] as a rationale to forgo treatment.  However, subsequent studies have shown that lack of self-awareness occurs more broadly across the spectrum of HD symptoms including dyskinesia [15] as well as cognitive, social, emotional and functional abilities [16], for which lack of awareness would not influence treatment decisions.

**Impact of chorea on motor dysfunction:** Studies have shown that chorea negatively impacts motor function for accuracy of movement, reaction time, and gait regulation [17] [18] [19].  However, HD is a mixed movement disorder with hypokinetic components that also negatively impact function.  So it is difficult to assess the relative contribution of chorea and hypokinetic components in total motor dysfunction, particularly in early or middle stages of adult onset disease when chorea is more prominent [20].   In contrast, bradykinesia begins early in adult onset disease [21], and correlates with decline in functional capacity over the entire course of disease [22]. Bradykinesia and rigidity are the dominant motor impairments in juvenile onset [23] and late-stage adult onset patients.  Late stage bradykinesia is a strong predictor of nursing home placement [24].

Although direct clinical research is lacking on chorea’s functional impact as an isolated symptom, even in those with severe chorea, there are several studies suggesting that chorea may play an independent role in functional disability. The suppression of chorea improved writing speeds by 50% [25]. Chorea score was a major factor in impairment of motor tracking, or of accurately completing a motor task [20]. In a retrospective analysis of CARE-HD study participants, severity of chorea independently correlated with functional disability among a subgroup least affected by non-chorea symptoms [26].  In very-early-disease subjects in the TRACK-HD study, motor scores were more highly associated with earliest functional decline in HD than cognitive or behavioral scores. Among the separate motor components, chorea scores showed the highest correlation with loss of function [27].  In later-disease subjects, severity of chorea was an independent predictor of fall frequency and gait disturbance in HD, and more highly correlated to number of falls than voluntary motor impairment [28].

  Survey results support a trial of drug treatment for motor component dysfunction when HD chorea causes difficulty performing motor tasks at the workplace, causes imbalance and falls, results in physical injury, or interferes with sleep.

**Impact of chorea on HD stigma:**  Though multiple factors are involved in HD stigma, chorea’s high visibility likely contributes to embarrassment and the social isolation of affected individuals and families. While there are no studies that have included quality of life measures in HD, related studies in other diseases suggest that stigma is high across the spectrum of chronic neurological disorders, particularly those that include visible motor components. Studies in epilepsy [29], multiple sclerosis [30], and Parkinson’s disease [31], indicate that stigma impacts quality of life in these disorders.  Studies of the high stigma which occurs in Tourette Syndrome (TS) and related motor tics may more closely approximate the stigma related to chorea in HD [32].  Further, in both HD and TS , the movement disorders are often mistaken for alcohol intoxication which in turn may further increase the level of stigma [33].

  Stigma in HD can likely be assessed by use of a scale validated for use in other chronic neurologic disease [34], which suggests the type of interview question that might be asked: “Because of my illness: (1) I feel embarrassed in social situations, (2) people avoid me, (3) strangers tend to stare at me, (4) people seem to be uncomfortable with me.”  The high frequency of stigma and discriminatory events that occur in individuals with a positive HD family history who have no symptoms has been documented in RESPOND-HD study [35].  It is unclear how substantially chorea of the affected family member contributes to stigmatization of unaffected family members. However, it may be a factor to consider in treatment decisions.

  Survey results strongly support drug treatment of chorea when stigma factors of embarrassment and social isolation affects the HD patient.  In practice this type of information should regularly be elicited from patients.  In contrast, there is minority support from the experts for treating the patient when HD stigma adversely affects family members.

**Chorea drug treatments:**  For the indications listed, this survey supports the use of drugs for the treatment of chorea with the goal of reducing symptom severity.  Though not addressed specifically in the survey, there is general agreement that pushing therapy to eliminate chorea will cause unacceptable levels of side effects.  Because each drug alternative has significant side effects, a careful evaluation of risk-benefit must be assessed for each individual prior to initiating drug therapy, and reassessed frequently both during drug titration and over the course of treatment.  Because chorea severity decreases with later progression of disease, the need for chorea treatment should be reassessed over time.  Further, the presence of comorbid symptoms should greatly influence the choice of drug for treating chorea.  

**Antipsychotic drug treatment for HD chorea:**  Though it is an off-label use, the first choice of most international respondents for treatment of chorea is an APD.  Importantly, APD use is strongly preferred when psychosis, depression, aggressive behaviors, or poor compliance are comorbid factors.  Although the use of second generation APDs was generally preferred, tiapride a first generation APD was a frequent European choice, and the lower cost of haloperidol, a first generation APD was a factor in treatment choice for several respondents in all geographic areas.  The choice of a specific APD was quite varied, which reflects the lack of evidence base to guide drug choice. 

 Only small studies and case reports are available; there are no large placebo-controlled or head-to-head comparison studies of APDs for treatment of HD chorea.  Chorea benefit was demonstrated in a small placebo-controlled trial of haloperidol [36].  Olanzapine has been used in small open label studies with variable benefit [37] [38] [39].  A few reports note benefit with risperidone [40] [41], and quetiapine [42].  Benefit was shown in small open label [43] and placebo-controlled [44] trials of tiapride, available in most countries in Europe.  A study with clozapine suggested minimal benefit, and significant side effects [45].  Studies with aripiprazole, a third generation APD, found a reduction in chorea similar to that observed with TBZ [46].  In the only study to compare functional capacity differences between a second generation APD (clotiapine) and TBZ for treatment of chorea [47], 38 participants were followed for a minimum of 2 years.  Loss of functional capacity was greater in the APD group (N=10) than that in the TBZ group (N=28).  However this study is retrospective and assignment to APD or TBZ was not randomized. This points out the important need to study and compare potential treatment-related functional losses in a prospective and randomized manner.    

Importantly, survey respondents reported frequent APD side effects, including sedation, Parkinsonism, apathy, cognitive decline, and less frequently, akathisia, metabolic syndrome, and tardive dyskinesia. Though not specifically addressed in the survey, side effects may vary according to specific drug choice (Table 5).  Because many of these symptoms occur as a consequence of disease progression, the contribution of drug side effects is difficult to assess.  If APDs are used to treat chorea, it is important to reassess dose requirements as the disease progresses.  Though dosage ranges for individual APDs were not addressed in the survey, respondents indicated that, in general, dosage less than that recommended for psychotic behaviors is preferred.  

**Table 5. Side effect profiles for APDs used for HD chorea.**

**Table:** 

**APD**	**Observed side effects**
olanzapine	weight gain, metabolic syndrome, sedation, dry mouth
risperidone	hyperprolactinemia, weight gain, Parkinsonism
quietapine	weight gain, sedation, akathisia, dry mouth
aripiprazole	arrhythmias, akathisia, sedation, Parkinsonism
haloperidol	sedation, Parkinsonism, akathisia, tardive dyskinesia
tiapride	sedation, Parkinsonism

**Tetrabenazine treatment for HD chorea:**  In North America, when choice was not influenced by other factors, TBZ was preferred almost as highly as APDs for chorea treatment.  In both North America and Europe, TBZ was a consistent alternative monotherapy choice.  TBZ is the only drug that the U.S. Federal Drug Administration has approved for the treatment of HD chorea.  TBZ is also available in Canada, Australia, Denmark, France, Germany, Ireland, Israel, Italy, New Zealand, Portugal, Spain, Switzerland, and the UK.  The efficacy of this drug was shown in a double-blinded, placebo-controlled study conducted by the HSG, in which drug was given as tolerated up to 100 mg/day [48].  A 3.5 point change in chorea score, or a 23.5% reduction from baseline was demonstrated in the treated group (chorea score range is 0 to 28), with about 50% of the treated group achieving a 6-point or greater improvement compared to 7% showing this level of response in the placebo group.  Importantly, however, more adverse events occurred in the treated group, including somnolence, insomnia, depressed mood, agitation, akathisia, and one suicide.  However, after stable dosing was achieved, there was no significant difference in adverse events between the treated and placebo groups. During the 80-week open-label extension phase of this study, a similar side effect profile emerged [49].  No quality of life issues were measured in this or any other TBZ chorea drug trial.  In the present survey, the perceived frequency of TBZ side effects, except for depression, was similar to or slightly lower than those perceived for APDs in treating chorea.  For HD in general, and particularly with the use of TBZ for HD chorea, it is imperative to have heightened caution regarding depression and suicide risk.  While studies suggest that risk of depression with TBZ is more likely to occur in those with pre-existing depression, it can also occur in those with no history of depression prior to TBZ [50].  As in the placebo controlled trial, survey responders reported more depression during the titration phase and first 2 months of stable dose TBZ treatment.  However, functional capacity appeared to be better preserved in a small number of patients treated with TBZ than in those treated by an APD over a 2 year time period [47].

The dosage after titration for the majority of participants in the HSG trial and open label extension was 50-75 mg/day, which was the dosage range preferred by the majority of survey respondents.  Lundbeck Inc. recommends and will provide reimbursement for testing CYP2D6 metabolizer levels prior to increasing TBZ dose above 50 mg/day.  In CYP2D6-poor metabolizers, concomitant use of strong inhibitors (paroxetine, fluoxetine, fluxoxamine) should be avoided, or TBZ dosage decreased.

**Benzodiazepine treatment for HD chorea:**  In a single study, high doses of clonazepam (up to 5.5 mg/day) were required to suppress chorea [51].  Survey respondents did not endorse use of BZDs as monotherapy, but thought BZDs an adjuctive therapy.

**Amantadine treatment for HD chorea:**  Two small placebo-controlled studies of amantadine for HD chorea provide conflicting results [52] [53]. When a meta-analysis of the combined trials was performed as part of the Cochrane review [11], the differences between treated and placebo groups did not reach significance.   Similarly, survey respondents disagreed about the use of this drug.  When chosen, amantadine’s most frequent use was as adjunctive therapy, though others thought its use inappropriate.

 Based on the results of this international expert survey, a clinical practice algorithm for the treatment of chorea in HD was constructed.  The authors do not mean to imply that following the steps most often chosen by experts will result in best outcomes. Treatment response varies greatly in HD, and is particularly hard to predict. The steps represented in the algorithm are meant to guide, not decide any individual's treatment. Only the clinician can address the complexities of any specific patient, where treatment must be tailored to fit individual needs.  

## **Algorithm**

 (Click on the figure below for a printable, single page view of the algorithm). 

**Figure:**
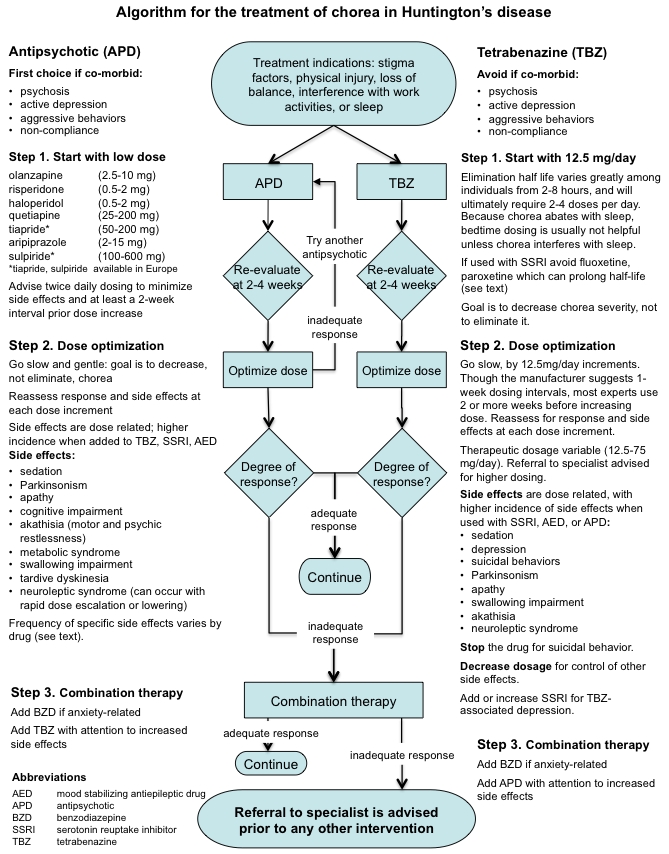

## **Conclusions**  

The expert respondents indicated a high level of agreement on treatment indications for chorea, including factors of stigma, impairments with work, sleep, safety and balance issues.  But they strongly disagreed about the first choice of drug, with a wide difference between experts from Europe, who preferred APDs, and those from North America and Australia, who chose equally between APDs and TBZ as a first treatment choice. APD usage for chorea was strongly favored by all groups when comorbid psychosis, aggression, or depression symptoms were present, or poor compliance suspected.  There was agreement that both APDs and TBZ are partially and variably effective, with similar side effect profiles for sedation, apathy, Parkinsonism, and akathisia, and that depression is a serious, but treatable adverse reaction for TBZ.  APDs and TBZ were sometimes used together to control combinations of symptoms, or for chorea inadequately controlled by either APDs or TBZ alone.  There was agreement that BZDs are not substantially effective as monotherapy, but agreement on their use as adjunctive therapy, particularly if anxiety is a comorbid factor. There was high level of disagreement about the use of amantadine, and agreement among users that this drug was not substantially effective. 

Survey results point out the important need for more study on the direct impact of chorea on motor function, and stigma factors in HD.   Results also show the pressing need for drug comparison study, both for efficacy and for side effect profiling. There is also need for studies to address the side effect functional consequences of APDs and TBZ when used to treat chorea. 

**Limitations: **Survey results are not a substitute for evidence-based study.  Instead, these results present treatment options based on a synthesis of opinions from a large group of experts.  As shown in this survey**, **practice patterns are influenced by the practice experience learned in subspecialty training and/or geographic location.  And although Lundbeck Inc, has created mechanisms for mitigating TBZ cost for low-income and non-insured individuals in the U.S, practice decisions were influenced by the cost and complexity of prescribing this drug in the United States.  Recall bias may also have occurred, with survey results limited by the accuracy of respondents’ recall, with potential for over- or underestimation of both drug efficacy and drug side effects.  This project received funding support in part by Lundbeck Inc., an arrangement that could introduce bias.  In an effort to limit this bias, HSG and EHDN core committee members and survey respondents had no knowledge of Lundbeck Inc. support during the survey process or data analysis.  Though we believe the survey questions were comprehensive, they did not cover every possibility and may have omitted other useful queries. Further, selection of experts surveyed was not systematic, but included those active in clinical research known to the authors. 

**Acknowledgements**

The authors thank those HD experts who shared knowledge and participated in this survey:  Karen Anderson, Tomasin Andrews, Kevin Biglan, Ann Catherine Bachoud-Levi, Jodi Cori-Bloom, Raphael Bonelli, Jean-Marc Burgunder, Jang Ho Cha, Edmond Chiu, Peter Como, Merit Cudkowicz. Matthias Dose, Richard Dubinsky, Alexandra Durr, Andy Feigen, Joaquim Ferreira, Mark Groves, Mark Guttman, Don Higgins, Stephen Hersch, Joseph Jankovic, Karl Kieburtz, Barry Kremer, Pierre Krystkowiak, Martin Kucharik, Blair Leavitt, Wayne Martin, Elizabeth McCusker, Marsha Nance, Michael Orth, Oksena Osuchowersky, Susan Perlman, Asa Petersen, Josef Priller, Ann Messer, Hugh Rickards, Raymund Roos, Adam Rosenblatt, Diana Rosas, Ann Rosser, Jan Roth, Kathleen Shannon, Burton Scott, Ira Shoulson, Shiela Simpson, Sarah Tabrizi, Erik van Dejin, Francis Walker, Eric Wexler, Vicki Wheelock, Daniel Zielonka. 

 The authors thank Dr. Richard Dubinsky who provided expert advice in survey creation, CHDI Foundation Inc. for expert advice and technical assistance, and Ann Covalt for editorial assistance.   

**Funding sources**

The Huntington’s Disease Society of America (HDSA), Huntington Society of Canada (HSC), European Huntington’s Disease Network (EHDN), and HD Drug Works (HDDW) provided funding for this project.  Support from Lundbeck Inc. was provided by a one time unrestricted grant to HDDW.  The combined funds from HDSA, HSC, EHDN, and HDDW provided stipend reimbursement for expert participation.  To prevent bias, experts were kept unaware of the Lundbeck Inc. grant. 

**Competing interests**

Dr. LaVonne Goodman received unrestricted grant and consultant fee support from Lundbeck, Inc. in 2009.

**Author roles**

- Drs. Jean-Marc Burgunder, Mark Guttman, and Susan Perlman shared equally in construction and review of survey questionnaire, review of data analysis and manuscipt.
- Dr. Dan van Kammen.  Expert advisor, review of data analysis and manuscript.
- Dr. Nathan Goodman.  Data analysis.
- Dr. LaVonne Goodman. Conception, organization and facilitator for execution of the project. Writing of the first draft and review of manuscript.

## References

[ref-491804511] A novel gene containing a trinucleotide repeat that is expanded and unstable on Huntington's disease chromosomes. The Huntington's Disease Collaborative Research Group. Cell. 1993 Mar 26;72(6):971-83. 8458085 10.1016/0092-8674(93)90585-e

[ref-1338823169] Wojaczyńska-Stanek K, Adamek D, Marszał E, Hoffman-Zacharska D. Huntington disease in a 9-year-old boy: clinical course and neuropathologic examination. J Child Neurol. 2006 Dec;21(12):1068-73. 17156701 10.1177/7010.2006.00244

[ref-2335521053] Lipe H, Bird T. Late onset Huntington Disease: clinical and genetic characteristics of 34 cases. J Neurol Sci. 2009 Jan 15;276(1-2):159-62. Epub 2008 Oct 31. 18977004 10.1016/j.jns.2008.09.029 PMC3140172

[ref-364916971] Foroud T, Gray J, Ivashina J, Conneally PM. Differences in duration of Huntington's disease based on age at onset. J Neurol Neurosurg Psychiatry. 1999 Jan;66(1):52-6. 9886451 10.1136/jnnp.66.1.52 PMC1736160

[ref-977735000] “Huntington’s Disease” Third Edition Oxford monographs on Medical Genetics, Clinical Neurology of Huntington’s disease; Barry Kramer: page 82

[ref-3815033692] Tabrizi SJ, Scahill RI, Durr A, Roos RA, Leavitt BR, Jones R, Landwehrmeyer GB, Fox NC, Johnson H, Hicks SL, Kennard C, Craufurd D, Frost C, Langbehn DR, Reilmann R, Stout JC; TRACK-HD Investigators. Biological and clinical changes in premanifest and early stage Huntington's disease in the TRACK-HD study: the 12-month longitudinal analysis. Lancet Neurol. 2011 Jan;10(1):31-42. Epub 2010 Dec 2. 21130037 10.1016/S1474-4422(10)70276-3

[ref-2766396660] Roos RA. Huntington's disease: a clinical review. Orphanet J Rare Dis. 2010 Dec 20;5(1):40. 21171977 10.1186/1750-1172-5-40 PMC3022767

[ref-3512788103] Bonelli RM, Wenning GK. Pharmacological management of Huntington's disease: an evidence-based review. Curr Pharm Des. 2006;12(21):2701-20. Review. 16842168 10.2174/138161206777698693

[ref-1029934370] Phillips W, Shannon KM, Barker RA. The current clinical management of Huntington's disease. Mov Disord. 2008 Aug 15;23(11):1491-504. Review. 18581443 10.1002/mds.21971

[ref-1404530426] Adam OR, Jankovic J. Symptomatic treatment of Huntington disease. Neurotherapeutics. 2008 Apr;5(2):181-97. Review. 18394562 10.1016/j.nurt.2008.01.008 PMC5084162

[ref-1756418420] Mestre T, Ferreira J, Coelho MM, Rosa M, Sampaio C. Therapeutic interventions for symptomatic treatment in Huntington's disease. Cochrane Database Syst Rev. 2009 Jul 8;(3):CD006456. Review. 19588393 10.1002/14651858.CD006456.pub2

[ref-3800699462] Groves M, van Duijn E, Anderson K, Craufurd D, Edmondson MC, Goodman N, van Kammen DP, Goodman L. An International Survey-based Algorithm for the Pharmacologic Treatment of Irritability in Huntington's Disease. PLoS Curr. 2011 Aug 30;3:RRN1259.http://knol.google.com/k/lavonne-goodman/an-international-survey-based-algorithm/p284k2gmahk5/10 21975525 10.1371/currents.RRN1259 PMC3166255

[ref-2892672850] Anderson K, Craufurd D, Edmondson MC, Goodman N, Groves M, van Duijn E, van Kammen DP, Goodman L. An International Survey-based Algorithm for the Pharmacologic Treatment of Obsessive-Compulsive Behaviors in Huntington's Disease. PLoS Curr. 2011 Sep 20;3:RRN1261.http://knol.google.com/k/lavonne-goodman/an-international-survey-based-algorithm/p284k2gmahk5/9 21947193 10.1371/currents.RRN1261 PMC3177175

[ref-527036229] Snowden JS, Craufurd D, Griffiths HL, Neary D. Awareness of involuntary movements in Huntington disease. Arch Neurol. 1998 Jun;55(6):801-5. 9626771 10.1001/archneur.55.6.801

[ref-757026283] Vitale C, Pellecchia MT, Grossi D, Fragassi N, Cuomo T, Di Maio L, Barone P. Unawareness of dyskinesias in Parkinson's and Huntington's diseases. Neurol Sci. 2001 Feb;22(1):105-6. 11487181 10.1007/s100720170066

[ref-57621794] Hoth KF, Paulsen JS, Moser DJ, Tranel D, Clark LA, Bechara A. Patients with Huntington's disease have impaired awareness of cognitive, emotional, and functional abilities. J Clin Exp Neuropsychol. 2007 May;29(4):365-76. 17497560 10.1080/13803390600718958

[ref-4059821308] Bilney B, Morris ME, Perry A. Effectiveness of physiotherapy, occupational therapy, and speech pathology for people with Huntington's disease: a systematic review. Neurorehabil Neural Repair. 2003 Mar;17(1):12-24. Review. 12645441 10.1177/0888439002250448

[ref-1910640615] Kim JS, Reading SA, Brashers-Krug T, Calhoun VD, Ross CA, Pearlson GD. Functional MRI study of a serial reaction time task in Huntington's disease. Psychiatry Res. 2004 May 30;131(1):23-30. 15246452 10.1016/j.pscychresns.2004.03.002

[ref-1580376886] Bilney B, Morris ME, Churchyard A, Chiu E, Georgiou-Karistianis N. Evidence for a disorder of locomotor timing in Huntington's disease. Mov Disord. 2005 Jan;20(1):51-7. 15390128 10.1002/mds.20294

[ref-46144837] Fenney A, Jog MS, Duval C. Bradykinesia is not a "systematic" feature of adult-onset Huntington's disease; implications for basal ganglia pathophysiology. Brain Res. 2008 Feb 8;1193:67-75. Epub 2007 Dec 8. 18177845 10.1016/j.brainres.2007.12.005

[ref-1440984839] Sánchez-Pernaute R, Künig G, del Barrio Alba A, de Yébenes JG, Vontobel P, Leenders KL. Bradykinesia in early Huntington's disease. Neurology. 2000 Jan 11;54(1):119-25. 10636136 10.1212/wnl.54.1.119

[ref-3953693232] Mahant N, McCusker EA, Byth K, Graham S; Huntington Study Group. Huntington's disease: clinical correlates of disability and progression. Neurology. 2003 Oct 28;61(8):1085-92. 14581669 10.1212/01.wnl.0000086373.32347.16

[ref-1482304605] Gonzalez-Alegre P, Afifi AK. Clinical characteristics of childhood-onset (juvenile) Huntington disease: report of 12 patients and review of the literature. J Child Neurol. 2006 Mar;21(3):223-9. 16901424 10.2310/7010.2006.00055

[ref-1641613920] Wheelock VL, Tempkin T, Marder K, Nance M, Myers RH, Zhao H, Kayson E, Orme C, Shoulson I; Huntington Study Group. Predictors of nursing home placement in Huntington disease. Neurology. 2003 Mar 25;60(6):998-1001. 12654967 10.1212/01.wnl.0000052992.58107.67

[ref-3212852021] McLellan DL, Chalmers RJ, Johnson RH. A double-blind trial of tetrabenazine, thiopropazate, and placebo in patients with chorea. Lancet. 1974 Jan 26;1(7848):104-7. 4130307 10.1016/s0140-6736(74)92338-1

[ref-4008078604] Frank S, Marshall F, Plumb S, Oakes D, Shoulson I, Kieburtz K, the CARE-HD Investigators of the Huntington Study Group: Functional decline due to chorea in Huntington's disease. Neurology 2004. , 62(suppl 5):

[ref-3361537390] Beglinger LJ, O'Rourke JJ, Wang C, Langbehn DR, Duff K, Paulsen JS; Huntington Study Group Investigators. Earliest functional declines in Huntington disease. Psychiatry Res. 2010 Jul 30;178(2):414-8. Epub 2010 May 15. 20471695 10.1016/j.psychres.2010.04.030 PMC3629818

[ref-1954541173] Grimbergen YA, Knol MJ, Bloem BR, Kremer BP, Roos RA, Munneke M. Falls and gait disturbances in Huntington's disease. Mov Disord. 2008 May 15;23(7):970-6. 18381643 10.1002/mds.22003

[ref-2837273438] Bandstra NF, Camfield CS, Camfield PR. Stigma of epilepsy. Can J Neurol Sci. 2008 Sep;35(4):436-40. Review. 18973059 10.1017/s0317167100009082

[ref-4003215995] Grytten N, Måseide P. 'When I am together with them I feel more ill.' The stigma of multiple sclerosis experienced in social relationships. Chronic Illn. 2006 Sep;2(3):195-208. 17007696 10.1177/17423953060020030101

[ref-4251035253] Chapuis S, Ouchchane L, Metz O, Gerbaud L, Durif F. Impact of the motor complications of Parkinson's disease on the quality of life. Mov Disord. 2005 Feb;20(2):224-30. 15384126 10.1002/mds.20279

[ref-56810755] Davis KK, Davis JS, Dowler L. In motion, out of place: the public space(s) of Tourette Syndrome. Soc Sci Med. 2004 Jul;59(1):103-12. 15087147 10.1016/j.socscimed.2003.10.008

[ref-304154860] Schomerus G, Holzinger A, Matschinger H, Lucht M, Angermeyer MC. [Public attitudes towards alcohol dependence]. Psychiatr Prax. 2010 Apr;37(3):111-8. Epub 2010 Feb 10. Review. German. 20148378 10.1055/s-0029-1223438

[ref-2604835401] Rao D, Choi SW, Victorson D, Bode R, Peterman A, Heinemann A, Cella D. Measuring stigma across neurological conditions: the development of the stigma scale for chronic illness (SSCI). Qual Life Res. 2009 Jun;18(5):585-95. Epub 2009 Apr 25. 19396572 10.1007/s11136-009-9475-1 PMC2875076

[ref-3464502826] Erwin C, Williams JK, Juhl AR, Mengeling M, Mills JA, Bombard Y, Hayden MR, Quaid K, Shoulson I, Taylor S, Paulsen JS; I-RESPOND-HD Investigators of the Huntington Study Group. Perception, experience, and response to genetic discrimination in Huntington disease: the international RESPOND-HD study. Am J Med Genet B Neuropsychiatr Genet. 2010 Jul;153B(5):1081-93. 20468061 10.1002/ajmg.b.31079 PMC3593716

[ref-169117834] Barr AN, Fischer JH, Koller WC, Spunt AL, Singhal A. Serum haloperidol concentration and choreiform movements in Huntington's disease. Neurology. 1988 Jan;38(1):84-8. 2962009 10.1212/wnl.38.1.84

[ref-3601945107] Bonelli RM, Mahnert FA, Niederwieser G. Olanzapine for Huntington's disease: an open label study. Clin Neuropharmacol. 2002 Sep-Oct;25(5):263-5. 12410058 10.1097/00002826-200209000-00007

[ref-2756234149] Squitieri F, Cannella M, Piorcellini A, Brusa L, Simonelli M, Ruggieri S. Short-term effects of olanzapine in Huntington disease. Neuropsychiatry Neuropsychol Behav Neurol. 2001 Jan;14(1):69-72. 11234911

[ref-1520650951] Jiménez-Jiménez FJ, de Toledo M, Puertas I, Barón M, Zurdo M, Barcenilla B. [Olanzapine improves chorea in patients with Huntington's disease]. Rev Neurol. 2002 Sep 16-30;35(6):524-5. Spanish. 12389168

[ref-965150351] Duff K, Beglinger LJ, O'Rourke ME, Nopoulos P, Paulson HL, Paulsen JS. Risperidone and the treatment of psychiatric, motor, and cognitive symptoms in Huntington's disease. Ann Clin Psychiatry. 2008 Jan-Mar;20(1):1-3. 18297579 10.1080/10401230701844802 PMC3806309

[ref-3402912648] Dallocchio C, Buffa C, Tinelli C, Mazzarello P. Effectiveness of risperidone in Huntington chorea patients. J Clin Psychopharmacol. 1999 Feb;19(1):101-3. 9934953 10.1097/00004714-199902000-00020

[ref-954422863] Alpay M, Koroshetz WJ. Quetiapine in the treatment of behavioral disturbances in patients with Huntington's disease. Psychosomatics. 2006 Jan-Feb;47(1):70-2. 16384811 10.1176/appi.psy.47.1.70

[ref-1550870098] Quinn N, Marsden CD. Tiapride in 12 Huntington's disease patients. J Neurol Neurosurg Psychiatry. 1985 Mar;48(3):292. 3156967 10.1136/jnnp.48.3.292 PMC1028279

[ref-4000115187] Deroover J, Baro F, Bourguignon RP, Smets P. Tiapride versus placebo: a double-blind comparative study in the management of Huntington's chorea. Curr Med Res Opin. 1984;9(5):329-38. 6241563 10.1185/03007998409109601

[ref-3195039226] van Vugt JP, Siesling S, Vergeer M, van der Velde EA, Roos RA. Clozapine versus placebo in Huntington's disease: a double blind randomised comparative study. J Neurol Neurosurg Psychiatry. 1997 Jul;63(1):35-9. 9221965 10.1136/jnnp.63.1.35 PMC2169648

[ref-2725153365] Brusa L, Orlacchio A, Moschella V, Iani C, Bernardi G, Mercuri NB. Treatment of the symptoms of Huntington's disease: preliminary results comparing aripiprazole and tetrabenazine. Mov Disord. 2009 Jan 15;24(1):126-9. 19170197 10.1002/mds.22376

[ref-3935003455] de Tommaso M, Serpino C, Sciruicchio V. Management of Huntington's disease: role of tetrabenazine. Ther Clin Risk Manag. 2011;7:123-9. Epub 2011 Mar 21. 21479143 10.2147/TCRM.S17152 PMC3071349

[ref-133394685] Huntington Study Group. Tetrabenazine as antichorea therapy in Huntington disease: a randomized controlled trial. Neurology. 2006 Feb 14;66(3):366-72. 16476934 10.1212/01.wnl.0000198586.85250.13

[ref-1697221181] Frank S. Tetrabenazine as anti-chorea therapy in Huntington disease: an open-label continuation study. Huntington Study Group/TETRA-HD Investigators. BMC Neurol. 2009 Dec 18;9:62. Erratum in: BMC Neurol. 2011;11:18. 20021666 10.1186/1471-2377-9-62 PMC2804668

[ref-4183537583] Kenney C, Hunter C, Mejia N, Jankovic J. Is history of depression a contraindication to treatment with tetrabenazine? Clin Neuropharmacol. 2006 Sep-Oct;29(5):259-64. 16960470 10.1097/01.WNF.0000228369.25593.35

[ref-2942537786] Peiris JB, Boralessa H, Lionel ND. Clonazepam in the treatment of choreiform activity. Med J Aust. 1976 Feb 21;1(8):225-7. 131236

[ref-742667921] O'Suilleabhain P, Dewey RB Jr. A randomized trial of amantadine in Huntington disease. Arch Neurol. 2003 Jul;60(7):996-8. 12873857 10.1001/archneur.60.7.996

[ref-3145761129] Heckmann JM, Legg P, Sklar D, Fine J, Bryer A, Kies B. IV amantadine improves chorea in Huntington's disease: an acute randomized, controlled study. Neurology. 2004 Aug 10;63(3):597-8; author reply 597-8. 15304616 10.1212/wnl.63.3.597

